# Supplementary material for: Phase II Clinical Study on Low-Intensity-Noise Tinnitus Suppression (LINTS) for Tinnitus Treatment
Source: Brain Sci. 2025 Nov 13;15(11):1222. doi: 10.3390/brainsci15111222 (PMC12650358; doi:10.3390/brainsci15111222)
Supplement: Supplementary file 1 [file brainsci-15-01222-s001.zip › brainsci-3930860-supplementary.pdf]

Table S1: Nonparametric comparison of patient subgroups with WB or NB stimulation.

group	parameter	subgroup	Chi^2 *or* Mann-Whitney U-test	Number *or* Median (interquartile range)
treatment only patient group (TO)	gender	WB	chi^2=5.71<br>p=0.02	male: 13<br>female: 5
		NB		male: 19<br>female: 11
	age	WB	U=714.0<br>p=0.67	53 a (43 a, 59 a)
		NB		50 a (34 a, 59 a)
	TF	WB	U=370.5<br>p=0.17	4 kHz<br>(4 kHz, 6 kHz)
		NB		6 kHz<br>(4 kHz, 8 kHz)
	TL	WB	U=776.0<br>p=0.71	5 dB SL<br>(2 dB SL, 8 dB SL)
		NB		6 dB SL<br>(2 dB SL, 8 dB SL)
Placebo plus treatment patient group (PT)	gender	WB	chi^2=1.14<br>p=0.29	male: 5<br>female: 5
		NB		male: 10<br>female: 4
	age	WB	U=37.0<br>p=0.06	62 a (58 a, 62 a)
		NB		52.5 a (41 a, 59 a)
	TF	WB	U=175.5<br>p=0.92	5 kHz<br>(3 kHz, 6 kHz)
		NB		6 kHz<br>(3 kHz, 8 kHz)
	TL	WB	U=152.5<br>p=0.49	5 dB SL<br>(3 dB SL, 10.5 dB SL)
		NB		4 dB SL<br>(3 dB SL, 9 dB SL)

Note: red colored p-values indicate a significant group difference.
